# Supplementary material for: The interactome of intact mitochondria by cross-linking mass spectrometry provides evidence for coexisting respiratory supercomplexes
Source: Mol Cell Proteomics. 2017 Dec 8;17(2):216–32. doi: 10.1074/mcp.RA117.000470 (PMC5795388; doi:10.1074/mcp.RA117.000470)
Supplement: Supplemental Data [file supp_RA117.000470_133922_0_supp_23978_fzffwf.zip › spectra_annotation/mito_spectra_annotation/254-1-7-1-7-1.pdf]

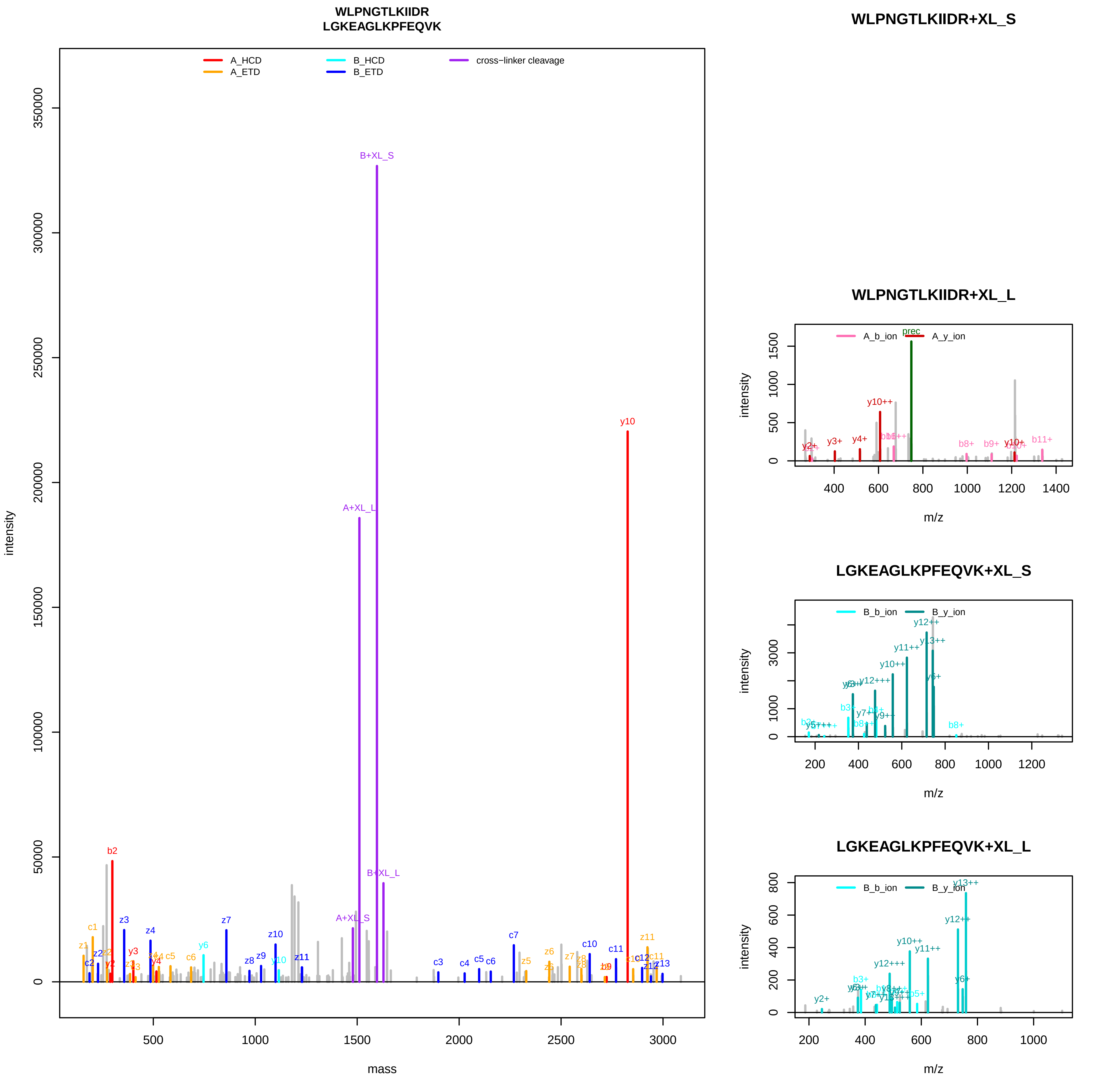
WLPNGTLKIIDR
LGKEAGLKPFEQVK
A_HCD
A_ETD
B_HCD
B_ETD
cross−linker cleavage
B+XL_S
A+XL_L
B+XL_L
A+XL_S
y10
b2
z3
z4
z7
z10
z11
c7
c10
c11
c12
z11
c11
z13
c3
c4
c5
c6
y6
z8
z9
z6
z7
z5
c1
z1
c2
z2
y3
y4
c5
c6
intensity
mass
0
50000
100000
150000
200000
250000
300000
350000
500
1000
1500
2000
2500
3000
WLPNGTLKIIDR+XL_S
WLPNGTLKIIDR+XL_L
A_b_ion
A_y_ion
prec
y10++
y2+
y3+
y4+
b8+
b9+
y10+
b11+
intensity
m/z
0
500
1000
1500
400
600
800
1000
1200
1400
LGKEAGLKPFEQVK+XL_S
B_b_ion
B_y_ion
y12++
y13++
y11++
y10++
y12+++
y6+
b3+
b8+
y9++
intensity
m/z
0
1000
3000
200
400
600
800
1000
1200
LGKEAGLKPFEQVK+XL_L
B_b_ion
B_y_ion
y13++
y12++
y10++
y11++
y12+++
y6+
b3+
y2+
intensity
m/z
0
200
400
600
800
200
400
600
800
1000
